# Slowdown of Translational Elongation in *Escherichia coli* under Hyperosmotic Stress

**DOI:** 10.1128/mBio.02375-17

**Published:** 2018-02-13

**Authors:** Xiongfeng Dai, Manlu Zhu, Mya Warren, Rohan Balakrishnan, Hiroyuki Okano, James R. Williamson, Kurt Fredrick, Terence Hwa

**Affiliations:** a Department of Physics, University of California at San Diego, La Jolla, California, USA; b School of Life Sciences, Central China Normal University, Wuhan, China; c Department of Microbiology and Ohio State Biochemistry Program, the Ohio State University, Columbus, Ohio, USA; d Department of Integrative Structural and Computational Biology, Department of Chemistry, the Skaggs Institute for Chemical Biology, the Scripps Research Institute, La Jolla, California, USA; Korea Advanced Institute of Science and Technology

**Keywords:** hyperosmotic stress, protein synthesis, ribosome content, translational elongation rate

## Abstract

In nature, bacteria frequently experience many adverse conditions, including heat, oxidation, acidity, and hyperosmolarity, which all tend to slow down if not outright stop cell growth. Previous work on bacterial stress mainly focused on understanding gene regulatory responses. Much less is known about how stresses compromise protein synthesis, which is the major driver of cell growth. Here, we quantitatively characterize the translational capacity of *Escherichia coli* cells growing exponentially under hyperosmotic stress. We found that hyperosmotic stress affects bacterial protein synthesis through reduction of the translational elongation rate, which is largely compensated for by an increase in the cellular ribosome content compared with nutrient limitation at a similar growth rate. The slowdown of translational elongation is attributed to a reduction in the rate of binding of tRNA ternary complexes to the ribosomes.

## IMPORTANCE

Hyperosmotic stress is a common stress condition confronted by *E. coli* during infection of the urinary tract. It can significantly compromise the bacterial growth rate. Protein translation capacity is a critical component of bacterial growth. In this study, we find for the first time that hyperosmotic stress causes substantial slowdown in bacterial ribosome translation elongation. The slowdown of translation elongation originates from a reduced binding rate of tRNA ternary complex to the ribosomes.

## INTRODUCTION

Bacteria frequently encounter various environmental stress conditions in their natural habitat. For *Escherichia coli*, the primary cause of the urinary tract infections, the bacterium needs to cope with the hyperosmotic environment existing in the bladder (1, 2). Hyperosmotic stress (e.g., a high concentration of salts or sugars) tends to draw water out of cells. In order to maintain its turgor pressure and growth, *E. coli* counters hyperosmotic stress by accumulating large amounts of osmolytes such as potassium ions, glutamate, and trehalose (3–6). Nevertheless, protein synthesis and bacterial growth are still adversely affected under hyperosmotic conditions (3, 6). Previous work mainly focused on elucidating the molecular interactions sensing and responding to hyperosmotic stress (7–9). However, it is not known how hyperosmotic stress affects the translation capacity, a critical component of bacterial growth (10).

In the present study, we quantitatively characterize the translational capacity of *E. coli* growing exponentially under hyperosmotic stress. We establish that hyperosmotic stress causes substantial slowdown in the translational elongation rate (ER), which is largely compensated for by an increase in the ribosome content compared to nutrient limitation. We further show that a reduced binding rate of tRNA ternary complexes (TCs) to ribosomes is the likely origin of the observed slowdown in translational elongation.

## RESULTS

**FIG 1:**
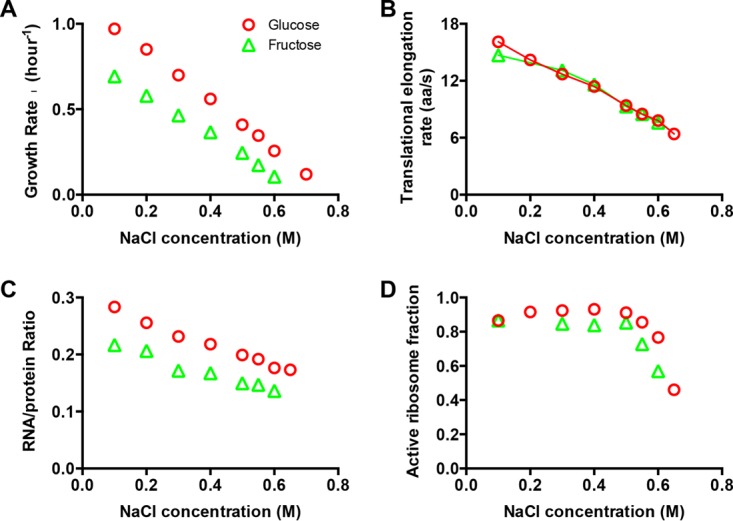
Growth and protein synthesis in hyperosmotic stress. (A) Growth rate versus sodium chloride (NaCl) concentration for strain NCM3722 growing in glucose and fructose MOPS-buffered minimal medium. (B) Translational elongation rate versus NaCl concentration for strain NCM3722 at different osmolarities. (C) Ribosome content versus NaCl concentration for strain NCM3722 at different osmolarities. (D) Active ribosome fraction versus NaCl concentration for strain NCM3722 at different osmolarities. Data points are the average of triplicate determinations. The standard deviations were around 5% to ~10% (approximately the size of the symbols).

We focused on wild-type *E. coli* K-12 strains growing exponentially under hyperosmotic conditions, achieved through supplementation of minimal growth medium with various concentrations of sodium chloride (see Materials and Methods). We first characterized the batch culture growth rate (λ), which is seen to decrease with increasing sodium chloride concentrations in both glucose and fructose minimal medium (Fig. 1A; see Fig. S1 in the supplemental material).

10.1128/mBio.02375-17.1 FIG S1  Growth curve of NCM3722 strain upon hyperosmotic stress. (A) Glucose minimal medium. (B) Fructose minimal medium. The growth medium was supplemented with different concentrations of sodium chloride to vary the osmolarity of the medium. For each condition, 5 to 10 data points at the OD_600_ range of 0.05 to ~0.5 were measured to obtain the exponential growth curve. Download FIG S1, PDF file, 0.1 MB. Copyright © 2018 Dai et al. 2018 Dai et al. This content is distributed under the terms of the Creative Commons Attribution 4.0 International license.

For exponentially growing bacteria, the total rate of protein synthesis depends on two crucial parameters, the translational elongation rate (ER) and the ribosome content (11, 12), and we characterized these two parameters under hyperosmotic conditions. Based on the classical LacZ induction assay (11, 13–15) (see Fig. S2 in the supplemental material), the ER was found to decrease steadily by 50% upon increasing hyperosmotic stress from 0.1 M to 0.6 M NaCl in both glucose and fructose minimal medium (red circles and green triangles, respectively, in Fig. 1B; see Table S1 in the supplemental material). To exclude the possibility that the results on ER are specific to LacZ protein, we employed two other methods—the LacZα fusion assay (11, 16) (see Fig. S3A-D in the supplemental material) and the classic pulse-chase labeling assay (11, 17) (see Fig. S3E-I in the supplemental material)—to measure the ERs of six other proteins: These ERs were found to decrease similarly at high osmolarity, and all the ER values obtained from different methods and for different proteins are quantitatively consistent with each other, as summarized in Fig. S3J in the supplemental material. We note that in principle, it is possible that the translational slowdown observed is a secondary effect of the slowdown of transcriptional elongation by RNA polymerase (RNAP) since ribosome follows RNAP during mRNA translation. However, by the pulse-chase labeling method (Fig. S3E-I), for a protein of 70 to 80 kDa (600 to ~700 amino acids [aa]) over half of the synthesized protein should be independent of the RNAP speed since the half-life of a normal mRNA is around 2 min (18). The consistency between the results of pulse-chase labeling and the LacZ and LacZα induction method therefore indicates that the observed slowdown is translational in origin.

10.1128/mBio.02375-17.2FIG S2 Raw data of LacZ induction assay and LacZα induction assay upon hyperosmotic stress. The LacZ induction assay was the first method used to measure the translational elongation rate of *E. coli*. (A) LacZ induction curve after IPTG addition for *E. coli* cells growing on different NaCl concentrations in glucose medium. The LacZ activity of the culture was plotted against the induction time after the addition of IPTG. (B) Schleif plot of the LacZ induction curve in panel A. The Schleif plot can be used to deduce the translation time of the first newly synthesized LacZ molecule after the addition of IPTG (11, 14, 15). The root square of the newly synthesized LacZ, $\sqrt{E(t)-E(0)}$, was plotted against the induction time. *E*(0) denotes the basal LacZ activity of the culture, and *E*(*t*) denotes the LacZ activity at specific time points after addition of IPTG. During the initial several minutes, $\sqrt{E(t)-E(0)}$ has a linear correlation with the induction time, and the *x* intercept of the linear line corresponds to the time needed for ribosome to translate a full-length LacZ molecule (*T*_first_). From panel B, *T*_first_ was significantly larger upon high osmolarity, suggesting a much slower translational elongation rate. (C). LacZα induction curve used for calibration of the time cost of initiation steps (*T*_init_) during the LacZ induction assay. The *T*_first_ estimated from the Schleif plot in panel B actually also contains several initiation steps, including IPTG penetration into cells, LacI derepression, RNA polymerase transcription initiation, and ribosome translation initiation. To calibrate the *T*_init_, we measured the induction curve of a small LacZα fragment (the N-terminal 90 amino acid residues of LacZ protein) (11, 16). Similar to previous studies, *T*_init_ remained constant at 10 s at different osmolarities. In this case, the translational elongation rate of LacZ, *k*, equals 1,024/(*T*_first_ − 10). Download FIG S2, PDF file, 0.2 MB.Copyright © 2018 Dai et al.2018Dai et al.This content is distributed under the terms of the Creative Commons Attribution 4.0 International license.

10.1128/mBio.02375-17.3 FIG S3  Raw data of the translational elongation rate obtained by other methods. (A to D) TufA-LacZα and FusA-LacZα induction assay upon hyperosmotic stress. The induction assay of the LacZα fusion protein was the second method used for measuring the translational elongation rate of *E. coli*. The basic principle of this method was exactly the same as the classical LacZ induction assay (16). The induction curves of two LacZα fusion proteins, TufA-LacZα (A) and FusA-LacZα (C), after IPTG induction were obtained based on LacZα complementation. The translation times of the first newly synthesized TufA-LacZα (B) and FusA-LacZα (D) were again obtained by Schleif plots. The translational elongation rates of TufA-LacZα and FusA-LacZα were equal to *L*/(T_first_ − 10), where *L* is the length of the LacZα fusion proteins (TufA-LacZα, 494 aa; FusA-LacZα, 804 aa). (E to I) Translational elongation rate upon hyperosmotic stress determined by pulse-chase radioactive labeling. The pulse-chase radioactive labeling was the third method used for measuring the translational elongation rate. The basic principle of this method was described by Dai et al. and Pedersen (11, 17). The translational elongation rates of four proteins at two sodium chloride concentrations were analyzed and are listed in the table in panel I. (J) Translational elongation rates obtained by different methods. In our study, the translational elongation rate was obtained by three methods: LacZ induction assay (Fig. 1B), LacZα induction assay (Fig. S3A to D), and pulse-chase radioactive labeling (Fig. S3E to I). The ER data from those three methods are plotted together for comparison. The ER data shown for the LacZα induction assay are the average of TufA-LacZα and FusA-LacZα data. The ER data shown for pulse-chase radioactive labeling are the average of the data from the four proteins. Download FIG S3, PDF file, 0.1 MB. Copyright © 2018 Dai et al. 2018 Dai et al. This content is distributed under the terms of the Creative Commons Attribution 4.0 International license.

**FIG 2:**
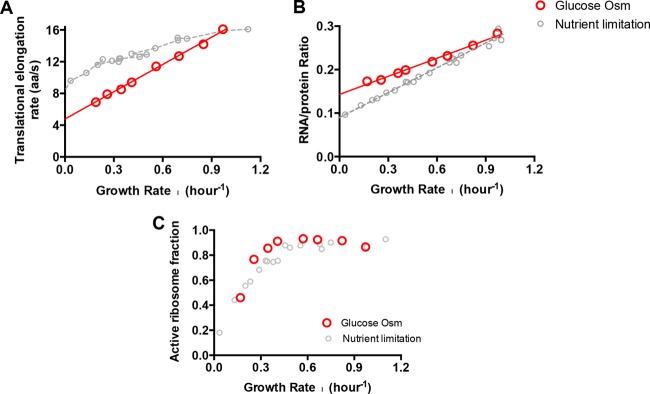
Comparison of translation parameters under hyperosmotic stress and under nutrient limitation. (A) Translational elongation rate. (B) Ribosome content. (C) Active ribosome fraction. The data points under hyperosmotic stress correspond to those in Fig. 1. The data points under nutrient limitation are replotted from data in the article by Dai et al. (11).

10.1128/mBio.02375-17.10TABLE S1 (A) Translational elongation rate of *E. coli* under different osmolarities. The data in glucose medium and fructose medium are shown in Fig. 1B. Determination of each value has been repeated for three times, and results are displayed as average ± standard error. (B) RNA/protein ratio of *E. coli* under different osmolarities. The data in glucose medium and fructose medium are shown in Fig. 1C. Total RNA and total protein values represent three repeat determinations, and results are displayed as average ± standard error. (C) Fraction of active ribosome of *E. coli* under different osmolarities. The data in glucose medium and fructose medium are shown in Fig. 1D. The fraction of active ribosome is calculated as follows: $f_{\text{active}}=N_{\text{Rb}}^{\text{active}}/N_{\text{Rb}}=(\lambda \cdot \sigma )/k\cdot (\text{R}/\text{P})$. The data for the translational elongation rate (*k*) are from table section A, and those for and R/P are from table section B. σ = *m*_rRNA_/(0.86 × *m*_aa_), where *m*_rRNA_ is the average molecular weight of rRNA (1,479,384) and *m*_aa_ is the average molecular weight of amino acid (113). (D) Translational elongation rate of *E. coli* upon chloramphenicol inhibition at a fixed high osmolarity. Wild-type *E. coli* NCM3722 cells were grown in medium of a fixed high osmolarity supplemented with different levels of chloramphenicol. The data are shown in Fig. 2A. Each value represents three repeat determinations, and values are displayed as average ± standard error. (E) RNA/protein ratio of *E. coli* upon chloramphenicol inhibition at a fixed high osmolarity. Wild-type *E. coli* NCM3722 cells were grown in medium of a fixed high osmolarity supplemented with different levels of chloramphenicol. The data are shown in Fig. 2B. Each value represents three determinations, and values are displayed as average ± standard error. Download TABLE S1, DOCX file, 0.1 MB.Copyright © 2018 Dai et al.2018Dai et al.This content is distributed under the terms of the Creative Commons Attribution 4.0 International license.

Another parameter crucial to protein synthesis is the abundance of the ribosomes, which can be readily deduced from the RNA/protein ratio (R/P) since the amount of rRNA is stoichiometrically related to the amount of ribosomal proteins (r-protein) and accounts for most (86%) of the RNA content (11, 12, 19, 20). As shown in Fig. S4, the proportionality between the R/P and the proteome fraction of r-protein still holds well under hyperosmotic stress, being the same as the case of nutrient limitation under normal osmolarity (11). Therefore, from here on, we use R/P as a proxy for the total ribosome content under hyperosmotic stress. It is seen to decrease by ~30% from 0.1 M to 0.6 NaCl (Fig. 1C).

10.1128/mBio.02375-17.4 FIG S4  Correlation between ribosome protein abundance and RNA/protein ratio under different osmolarity and nutrient limitation conditions. The RNA/protein ratio has long been used to represent the bacterial ribosome content since the amount of rRNA accounts for 86% of total RNA and is stoichiometrically proportional to the ribosomal protein (r-protein) content under different nutrient conditions (11, 20). To demonstrate this is still the case for *E. coli* hyperosmotic stress, we used quantitative mass spectrometry to quantify the abundance of all the r-proteins (35). The sum of the proteome fractions of all the r-proteins for *E. coli* growing in glucose medium at different osmolarities was plotted against the RNA/protein ratio. From the plot, we can find that the proportionality between r-protein abundance and RNA/protein ratio still holds under hyperosmotic stress, being the same as the case of nutrient limitation. Download FIG S4, PDF file, 0.1 MB. Copyright © 2018 Dai et al. 2018 Dai et al. This content is distributed under the terms of the Creative Commons Attribution 4.0 International license.

Since the cellular ribosomal content and ER depend intimately on the growth rate (11, 21, 22), while hyperosmolarity reduces cell growth (Fig. 1A), we must take the altered growth rate into account when evaluating the effect of hyperosmolarity on these quantities. We will do so by comparing them to effects due to nutrient limitation at the same growth rate, since we have recently characterized systematically the relationship between ER, R/P, and growth rate under nutrient limitation (11). In Fig. 2, we plotted the ER and R/P with growth rate for both hyperosmotic stress (red) and nutrient limitation (gray). The drop in ER under hyperosmotic stress is nearly 2-fold steeper than the drop under nutrient limitation (Fig. 2A), while the drop in R/P under hyperosmotic stress is less than that under nutrient limitation (Fig. 2B). Thus, cells under hyperosmotic stress have somewhat higher ribosome content than cells growing at the same rate under nutrient limitation, likely a result of a compensatory response to the larger drop in ER.

We next estimated the fraction of active ribosomes. For exponentially growing bacteria with negligible protein degradation, mass balance leads to the following equation (11, 23):

(1) $$\lambda \;\times \;N_{\text{aa}}=k\times N_{\text{Rb}}^{\text{active}}$$

*N*_aa_ is the number of amino acids contained in total proteins in a culture and *k* is the translational elongation rate. $N_{\text{Rb}}^{\text{active}}$, the number of actively translating ribosomes in a culture, can be calculated since the other three quantities in equation 1 have been directly measured in our study. We can further obtain the fraction of active ribosomes, $f_{\text{active}}=N_{\text{Rb}}^{\text{active}}/N_{\text{Rb}}$, since the total number of ribosomes in a culture, *N*_Rb_, is known through the RNA/protein ratio (11, 20). The result for *f*_active_ under hyperosmotic stress is shown in Fig. 1D. It is almost constant for [NaCl] at <0.5 M, but drops significantly at higher osmolarity. When plotted against the growth rate, *f*_active_ was seen to be at a constant high value (~85%) from moderate to fast growth (λ > 0.4/h), but dropped significantly for slower growth (Fig. 2C). Comparing to the growth rate dependence exhibited under nutrient limitation (gray symbols), we see that the two have similar trends, with the active fraction under hyperosmolarity being only slightly (<10%) larger than that under nutrient limitation. Thus, the loss in ER under hyperosmolarity is largely compensated for by increases in the amount of the ribosomes.

Since ER decreases remarkably under hyperosmotic stress, we next investigated the origin of the reduction in ER by revisiting a recently established quantitative model of translation elongation (11). In this coarse-grained model, the aminoacyl-tRNA/EF-Tu/GTP ternary complex (TC) is treated as the substrate of the ribosome (24). In this scenario, the elongation rate, *k*, has a Michaelis-Menten dependence on the TC concentration (11):

(2) $$\frac{1}{k}=\frac{1}{k_{\text{on}}\times \left[{\text{TC}}_{\text{eff}}\right]}+\frac{1}{k_{\text{elong}}}$$

*k*_elong_ is the maximal rate of translation elongation, *k*_on_ is the on rate of TC-ribosome binding, and [TC_eff_] is the effective concentration of TCs. The TC concentrations are difficult to quantify. Recently, it has been shown that the RNA/protein ratio could conveniently be used as a proxy of [TC_eff_] under nutrient limitation and translation inhibition (11), since the ribosome abundance was proportional to the EF-Tu abundance and tRNA abundance, and the charged fraction of tRNA was approximately constant under those growth conditions. The relation between [TC_eff_] and R/P could be described as [TC_eff_] = *C* × (R/P), where the proportionality constant, *C*, was estimated to be ~31 µM under normal osmolarity (11). The above relation leads to a Michaelis-Menten relation between ER and R/P and has been validated under both nutrient limitation and translation inhibition (11) (see Fig. S5 in the supplemental material).

10.1128/mBio.02375-17.5 FIG S5  The Michaelis-Menten relation between translational elongation rate and RNA/protein ratio (R/P) upon translational inhibition by chloramphenicol. In our recently established coarse-grained model of translational elongation (2), the aminoacyl-tRNA/EF-Tu/GTP ternary complex (TC) is treated as the substrate of the ribosome. In this scenario, ER has a Michaelis-Menten dependence on the TC concentration. We further found that R/P could be used as a proxy of TC concentration because of the constant proportionality between ribosome abundance and TC components under both nutrient limitation and chloramphenicol inhibition. In this case, the translational elongation rate was found to increase upon Cm inhibition for cells growing under several nutrient conditions at normal osmolarity (A) because of the increased TC concentrations caused by chloramphenicol (reflected by R/P) (B). Overall, the relation between ER and R/P at nutrient limitation (black circles in panel C) and Cm inhibition (cyan circles in panel C, which include all the colored data points of panels A and B) at normal osmolarity could be described by the same Michaelis-Menten relation (the black fit line). Data points are replotted from reference 11. RDM, rich defined medium. Download FIG S5, PDF file, 0.1 MB. Copyright © 2018 Dai et al. 2018 Dai et al. This content is distributed under the terms of the Creative Commons Attribution 4.0 International license.

Here under hyperosmotic stress, we found the RNA/protein ratio to be still proportional to EF-Tu (see Fig. S6 in the supplemental material) and the charged fraction of tRNA to be still approximately constant (green triangles in Fig. S7), similar to the case under nutrient limitation and translation inhibition (black circles and red triangles, respectively, in Fig. S7). However, because the cytoplasmic water content drops significantly under hyperosmotic stress (3, 6), the constant *C* drops with increasing NaCl concentration, leading to increased ternary complex concentration, [TC_eff_] (Fig. S8).

10.1128/mBio.02375-17.6 FIG S6  Correlation between EF-Tu protein abundance and RNA/protein ratio at different osmolarities. EF-Tu is a key component in the tRNA ternary complex, which is the substrate of protein translation. We measured its proteome abundance at different osmolarities. As shown in panel (A), the EF-Tu abundance was found to be proportional to the ribosome content as denoted by RNA/protein ratio. For panel B, we calculated the EF-Tu/ribosome ratio at different osmolarities. The EF-Tu number was calculated by using the total amount of EF-Tu (obtained using the total cellular protein amount times the proteome fraction of EF-Tu) to divide its molecular weight, 43,238. The number of ribosomes was calculated using the amount of cellular rRNA (86% of total RNA) to divide the molecular weight of rRNA, 1,479,384 (11, 12, 20). We found that the EF-Tu/ribosome was constant at a value of ~6. Download FIG S6, PDF file, 0.03 MB. Copyright © 2018 Dai et al. 2018 Dai et al. This content is distributed under the terms of the Creative Commons Attribution 4.0 International license.

10.1128/mBio.02375-17.7 FIG S7  The charged fractions of six tRNA species at different osmolarities. The aminoacyl-tRNA tRNA (charged tRNA) is the key component of the tRNA ternary complex. It has been well known that total tRNA is coregulated and proportional to the rRNA content. We further measured the charged fractions of six tRNA species by Northern blotting. (A) Typical image of the Northern blotting data of tRNA^Arg2^ at different osmolarities. There are two bands in the image, denoting charged tRNA and uncharged tRNA, respectively. The location of uncharged tRNA can be indicated by treating the total RNA with base (Tris-HCl) to convert all the tRNA into uncharged tRNA. We measured the charged fractions of six tRNA species upon hyperosmotic stress: (B) tRNA^Glu2^, (C) tRNA^Leu1^, (D) tRNA^Leu3^, (E) tRNA^Arg2^, (F) tRNA^Asp1^, and (G) tRNA^Gly3^. The data upon hyperosmotic stress (cyan triangles, including 0.1, 0.4, and 0.6 M NaCl in glucose medium) were plotted together with those upon nutrient limitation (dark circles) and chloramphenicol (Cm) inhibition (red triangles, including 0, 4, and 8 µM Cm in glucose medium plus 0.1 M NaCl) (11). For all six tRNA species, charged tRNA remains nearly constant under all growth conditions. Download FIG S7, PDF file, 0.2 MB. Copyright © 2018 Dai et al. 2018 Dai et al. This content is distributed under the terms of the Creative Commons Attribution 4.0 International license.

10.1128/mBio.02375-17.8 FIG S8  Deduction of the effective concentration of ternary complex, [TC_eff_], under hyperosmotic stress. (A) [TC_eff_] = *C* × (R/P). The cytoplasm water content drops substantially under hyperosmotic stress. Since the constant *C* is proportional to the cytoplasmic water amount (water weight/dry weight [W/DW]) (see definition in reference 11, *C* also decreases together with water content under hyperosmotic stress. The data for the cytoplasm water amount are from the study by Cayley et al. (6), in which their K-12 strain was grown in the same MOPS glucose minimal medium under different NaCl concentrations as used in our study. (B) [TC_eff_] under hyperosmotic stress. The R/P values correspond to data shown in Fig. 1B. [TC_eff_] is deduced based on [TC_eff_] = *C* × (R/P), where the data of constant *C* are taken to be the red symbols in panel A. Download FIG S8, PDF file, 0.04 MB. Copyright © 2018 Dai et al. 2018 Dai et al. This content is distributed under the terms of the Creative Commons Attribution 4.0 International license.

**FIG 3:**
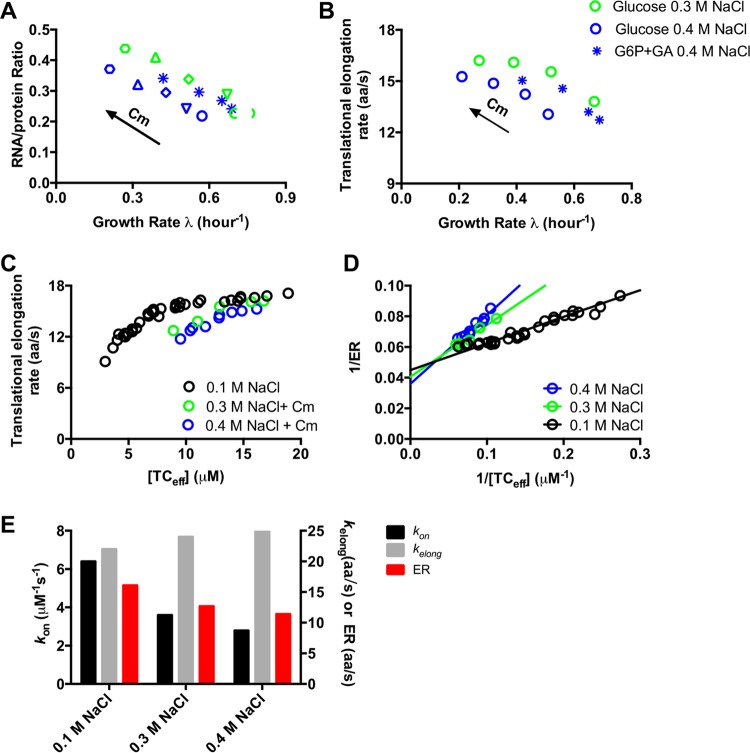
Michaelis-Menten correlation between translational elongation rate and ribosome content in a fixed high osmolarity. (A) RNA/protein ratio (R/P) in Cm inhibition under a fixed high osmolarity in both glucose medium and glucose-6-phosphate–gluconate medium. (B) Translational elongation rate in chloramphenicol (Cm) inhibition for a fixed high osmolarity (green, 0.3 M NaCl; blue, 0.4 M NaCl). (C) Correlation between translational elongation rate and the effective concentration of ternary complex ([TC_eff_]). [TC_eff_] data were obtained based on [TC_eff_] = *C* × (R/P), where the values of *C* were 31 (from reference 11), 25, and 22 µM in 0.1, 0.3, and 0.4 M NaCl, respectively (Fig. S8). ER data for 0.1 M NaCl include both nutrient limitation and Cm inhibition, as shown in Fig. S5. (D) Lineweaver-Burk plot of panel C at different osmolarities. The slope of the linear correlation denotes 1/*k*_on_, and the *y* intercept denotes 1/*k*_elong_. (E) Summary of ER, *k*_on_, and *k*_elong_ at different osmolarities. From the plot of panel D, the *k*_on_ values in 0.1, 0.3, and 0.4 M NaCl are 6.4, 3.6, and 2.8 µM^−1^ s^−1^, respectively, and the *k*_elong_ values in 0.1, 0.3, and 0.4 M NaCl are 22, 24, and 25 aa/s, respectively.

According to equation 2, the drop in ER may be due to changes in [TC_eff_], *k*_on_, and/or *k*_elong_. Since hyperosmotic stress reduces the cytoplasmic water amount, [TC_eff_] even increased under these conditions (Fig. S8). Therefore, the decrease in ER is likely caused by the decrease in *k*_on_ and/or *k*_elong_. To distinguish these two factors, we adopted a recently described approach (11) to vary the TC concentration by growing cultures with different amounts of the translational inhibitor chloramphenicol (Cm), here at various fixed osmolarities. In normal osmolarity, ER was found to increase upon Cm inhibition because of the increased [TC_eff_] caused by Cm (Fig. S5) (11). Under those conditions, the relationship between ER and [TC_eff_] under both nutrient limitation and Cm inhibition could be uniformly described by a Michaelis-Menten relation (Fig. S5C). Similarly, Fig. 3A shows that for different fixed high osmolarity, R/P significantly increased in Cm treatment, indicating a concomitant increase in [TC_eff_], as found in normal osmolarity (Fig. S5B). The increased [TC_eff_], upon addition of Cm at 0.3 and 0.4 M NaCl, is accompanied by increases in ER (Fig. 3B), again as is known under normal osmolarity (Fig. S5A) (11). The correlation between ER and [TC_eff_] under different fixed external osmolarities could be analyzed within the Michaelis-Menten framework (11) (Fig. 3C). The corresponding Lineweaver-Burk plots of equation 2 (Fig. 3D) reveal a vertical intercept that is almost invariant at high osmolarity, giving *k*_elong_ = 24 ± 2* *aa/s. On the other hand, the slope (1/*k*_on_) clearly increased, by ~60% at 0.4 M NaCl compared with 0.1 M NaCl (Fig. 3E). Our results thus suggest that hyperosmotic stress reduces the steady-state translational elongation rate by inhibiting the on rate between the ternary complexes and the ribosomes, from *k*_on_ = 6.4 µM^−1^ s^−1^ to 2.8 µM^−1^ s^−1^ for the range of external osmolarities from 0.28 osM (0.1 M NaCl) to 0.83 osM (0.4 M NaCl) (Fig. 3E).

## DISCUSSION

Hyperosmolarity is a common stress condition encountered by *E. coli* (1, 2). Our work has demonstrated that the translational elongation rate slowed down by 2-fold under hyperosmolarity compared to nutrient limitation over the same growth rate range. The Michaelis-Menten correlation analysis indicates that this drop occurs due to a 2-fold reduction in the binding of tRNA ternary complex to the ribosome. This may be caused by different mechanisms that are not mutually exclusive. For example, it may originate from the dramatically increased intracellular potassium pool under hyperosmolarity (3, 6, 25), which may negatively affect the equilibrium and kinetics of the interaction between tRNA ternary complex and the ribosome. Another possibility is macromolecular crowding. It has been proposed that even under normal osmolarity, the slow diffusion of tRNA ternary complex in the crowded bacterial cytoplasm imposes a physical limit on the speed of translational elongation by ribosomes (24, 26). Since the cytoplasm becomes even more crowded under hyperosmotic stress due to reduction in water content, the diffusion of tRNA ternary complex may become even slower, leading to the reduced on rates for TC-ribosome binding (27–29). It was found previously that the diffusion of green fluorescent protein (GFP) slowed down under hyperosmolarity (28). A comparison of the deduced *k*_on_ and GFP diffusion coefficient in cells adapted to hyperosmolarities found the drop of GFP diffusion to be about half of that of *k*_on_ over the range of osmolarity where both data are available (0.3 to ~1.0 osM) (Fig. S9). Since the tRNA ternary complex (70 kDa; linear physical dimension, ≈12 nm; PDB accession no. 1B23) (30) is much larger physically than GFP (30 kDa; linear physical dimension, ≈4.7 nm; PDB bank accession no. 1GFL) (31, 32), it is possible that its diffusion rate is more severely affected by high osmolarity than GFP in the crowded cytoplasm (28, 29).

10.1128/mBio.02375-17.9 FIG S9  Comparison between *k*_on_ and GFP diffusion coefficient under different osmolarities. It was found that the diffusion of GFP slowed down mildly for cells growing exponentially under hyperosmotic stress (28). We plot here the deduced changes in *k*_on_ (red circles) together with the data reported by Konopka et al. (28) on the GFP diffusion coefficient of adapted cells (blue circles) for direct comparison. Download FIG S9, PDF file, 0.03 MB. Copyright © 2018 Dai et al. 2018 Dai et al. This content is distributed under the terms of the Creative Commons Attribution 4.0 International license.

The active fraction of the ribosome decreased by ~2-fold at very slow growth under hyperosmolarity, similar to previous observations made under nutrient-poor conditions (Fig. 2C). Possible origins of ribosome inactivation include sequestration of ribosomes by ribosome-inactivating proteins (33), inhibition of the translational initiation (34), and abortion of translation elongation due to stalled ribosomes (11). In the future, it will be interesting to investigate whether there exists a common mechanism that reduces the active ribosome fraction under different kinds of adverse conditions.

Since the elongation rate dropped significantly (~50%) under hyperosmolarity compared to nutrient limitation (Fig. 2A), it is tempting to attribute the cause of growth slowdown to the reduced ER. However, reduction in ER itself does not necessarily need to result in reduction in growth. For example, a previously characterized ribosome mutant strain (streptomycin resistant [Sm^r^]) also had an ~50% reduction in ER compared to its wild type counterpart, but grew at a rate similar to the wild-type strain in minimal medium. It turned out that the drop in ER in the Sm^r^ strain was compensated for by increased active ribosome fraction (20). For the case of hyperosmolarity, we found the reduced ER to be largely compensated for by the moderate increase of the ribosome content (Fig. 2B), in the sense that a similar active ribosome fraction can account for the observed protein synthesis flux under high-osmolarity and nutrient-poor conditions (Fig. 2C). The compensation in ribosome content can be a burden for cell growth, as shown by previous studies overexpressing other useless proteins (20, 35). However, given the moderate increase in ribosome content, it seems unlikely that translational slowdown itself is the major cause of the substantial growth slowdown encountered. Nevertheless, the slowdown in translation established here is surely one of the important physiological problems cells have to deal with when growing under hyperosmotic conditions and must be confronted within a grand understanding of adaptation to osmotic stress.

## MATERIALS AND METHODS

### Strains.

The strains used in the study include the wild-type *E. coli* K-12 NCM3722 strain and its derivative, NQ1468, for measurement of the LacZα induction kinetics (11), the FL-2 strain for measurement of the translational elongation rate of FusA-LacZα protein, and the FL-3 strain for measurement of the translational elongation rate of TufA-LacZα protein.

To construct the FL-2 and FL-3 strains, the cassettes containing Ptet-*lacZ*ω/Plac-*fusA-lacZ*α and Ptet-*lacZ*ω/Plac-*tufA-lacZ*α in the pKUT15 series were digested by AvrII, gel purified with a kit (Tsingke Biological Technology), and inserted into the SpeI (the isocaudarner of AvrII) site of low-copy pBBR plasmid to obtain pFL-fusA and pFL-tufA vectors, respectively. A *lacZ*-deficient NCM3722 strain, FL1, was then made through transfer of the Δ*cynX782*::*kan* Δ*lacZ4787*(::*rrnB-3*) allele in strain JW0332 (Keio Collection in CGSC) to the wild-type NCM3722 strain through P1 transduction (16). The pFL-fusA and pFL-tufA vectors were transformed into the FL1 strain to obtain the FL-2 and FL-3 strains, respectively.

### Growth medium.

The growth medium used in this study was MOPS (morpholinepropanesulfonic acid)-buffered medium (pH 7.4) containing 40 mM MOPS (Coolaber, Beijing), 4 mM Tricine, 0.1 mM FeSO_4_, 0.276 mM Na_2_SO_4_, 0.5 µM CaCl_2_, 0.523 mM MgCl_2_, and micronutrients as detailed by Cayley et al. (3). The carbon sources added as supplements to the medium were 0.2% glucose or 0.2% fructose. The nitrogen source was 10 mM NH_4_Cl. The medium was supplemented with different concentrations of NaCl to vary the osmolarity. Different concentrations of chloramphenicol were added to achieve different extents of translational inhibition.

### Cell growth.

The cell growth experiments were performed in a 37°C water bath shaker. A standard cell growth experiment series contains three steps: seed culture, preculture, and the final experimental culture. Cells from a fresh colony in an LB solid agar plate were inoculated into LB medium (Solarbio Life Sciences) or LB medium plus 0.3 M NaCl (for culture that finally grew under high osmolarity) as seed culture. After several hours, cells were transferred into MOPS medium (the same as the final experimental medium) for growth overnight as a preculture. The next day, the cells in precultures were transferred to fresh MOPS medium at an initial optical density at 600 nm (OD_600_) of ~0.015 as the final experimental culture. To obtain the growth rate of the culture, 5 to 10 OD_600_ data points were recorded within the OD_600_ range of 0.05 to 0.5 to obtain the exponential growth curve.

### Measurement of translation elongation rate.

The translational elongation rates of the ribosome in this study were independently measured by the following three methods. (i) The first method was the LacZ induction assay, where the translation time of the first newly synthesized LacZ after IPTG (isopropyl-β-d-thiogalactopyranoside) induction, *T*_first_, was obtained through the Schleif plot of the LacZ induction curve. The time cost of the initiation steps during LacZ induction was calibrated by the LacZα induction curve and found to be constant at 10 s. The translational elongation rate, *k*, equals 1,024/(*T*_first_ − 10). (ii) The second method was the LacZα fusion protein induction assay, where the translation time of the first newly synthesized LacZα fusion protein (FusA-LacZα or TufA-LacZα) was similarly obtained by Schleif plot of the induction curve. The translational elongation rate, *k*, equals the length of the LacZα fusion protein, *L*, divided by *T*_first_ − 10. (iii) The third method was pulse-chase radioactive labeling. Pulse-chase radioactive labeling with a ^35^S-labeled methionine incorporation assay was used to obtain the translational elongation rate of another four proteins.

The detailed processes of the three methods described above were the same as described by Dai et al. (11).

### Total RNA quantification.

Total RNA quantification was performed similarly to the method described by You et al. (36). Briefly, 1.5 ml cell culture during exponential phase (OD_600_ of ~0.4) was rapidly collected by centrifugation, and the cell pellet was frozen in dry ice and stored at −80°C before measurement. Cell pellets were first washed twice with 0.6 ml cold 0.7 M perchloric acid (HClO_4_) and then digested by 0.3 ml of 0.3 M KOH for 1 h at 37°C with occasional mixing. The cell extract was further neutralized by 0.1 ml of 3 M HClO_4_, and the reaction mixture was centrifuged to collect the supernatant. The precipitate was then washed twice with 0.55 ml 0.5 M HClO_4_, and the supernatants were combined to give a 1.5-ml supernatant. The supernatant was then centrifuged to remove nonvisible precipitate and further measured for its absorbance at 260 nm (*A*_260_). The total amount of RNA (*R*) was calculated by the formula *R* (µg/ml/OD_600_) = *A*_260_ × 31/OD_600_.

### Total protein quantification.

Total protein quantification is based on the biuret method as described by You et al. (36). Briefly, 1.8 ml cell culture during the exponential phase (OD_600_ of ~0.4) was rapidly collected by centrifugation. The cell pellet was washed with NaCl solution of similar osmolarity to the growth medium and finally suspended in 0.2 ml of the same NaCl solution. The sample was frozen in dry ice and stored at −80°C before measurement. The thawed cell sample was digested with 0.1 ml 3 M NaOH and further heated at 100°C for 5 min before cooling down to room temperature. The cell mixture was then added to 0.1 ml of 1.6% CuSO_4_, shaken thoroughly, and allowed to stand for 5 min at room temperature. The reaction mixture was centrifuged, and the supernatant was measured for its absorbance at 555 nm. A similar experimental process was applied simultaneously to a series of bovine serum albumin (BSA) standards to obtain a standard curve. The amount of bacterial protein was obtained based on the BSA standard curve.

### Measurement of ribosome protein and EF-Tu abundance by quantitative proteomics.

To obtain the abundance of ribosome proteins and EF-Tu of *E. coli* growing under different osmolarities, we performed a quantitative mass spectrometry experiment on the cells to obtain proteome information. The detailed process is the same as that described by Hui et al. (35). The same procedure was applied to the MG1655 strain growing in the MOPS glucose medium as that described by Li et al. (37) as the reference condition for which the abundances of all the individual proteins have already been determined by ribosome profiling; the absolute abundances of EF-Tu and each ribosome protein of NCM3722 strain growing under different osmolarities were obtained through calibration with the reference condition.

### Measurement of aminoacyl-tRNA fraction.

The aminoacyl-tRNA (charged tRNA) fraction was measured by Northern blotting under acidic conditions (38). The extraction of total aminoacyl-tRNA under acidic conditions and the subsequent acidic gel electrophoresis and tRNA hybridization processes were performed the same as described by Janssen et al. (38). The tRNA-specific probes are listed in the article by Dong et al. (39).

## References

[1] Ross DL, Neely AE 1983 Textbook of urinalysis and body fluids. Appleton-Century-Crofts, New York, NY.

[2] Durack DT. 1987 Detection, prevention and management of urinary tract infections. Lea & Febiger, Philadelphia, PA.

[3] Cayley S, Lewis BA, Guttman HJ, Record MT Jr 1991 Characterization of the cytoplasm of Escherichia coli K-12 as a function of external osmolarity. Implications for protein-DNA interactions in vivo. J Mol Biol 222:281–300. doi:10.1016/0022-2836(91)90212-O. 1960728

[4] Csonka LN 1989 Physiological and genetic responses of bacteria to osmotic stress. Microbiol Rev 53:121–147. 2651863 10.1128/mr.53.1.121-147.1989 PMC372720

[5] Record MT Jr, Courtenay ES, Cayley DS, Guttman HJ 1998 Responses of E. coli to osmotic stress: large changes in amounts of cytoplasmic solutes and water. Trends Biochem Sci 23:143–148. doi:10.1016/S0968-0004(98)01196-7. 9584618

[6] Cayley S, Record MT Jr 2003 Roles of cytoplasmic osmolytes, water, and crowding in the response of Escherichia coli to osmotic stress: biophysical basis of osmoprotection by glycine betaine. Biochemistry 42:12596–12609. doi:10.1021/bi0347297. 14580206

[7] Hengge-Aronis R 1996 Back to log phase: sigma S as a global regulator in the osmotic control of gene expression in Escherichia coli. Mol Microbiol 21:887–893. doi:10.1046/j.1365-2958.1996.511405.x. 8885260

[8] Wood JM 1999 Osmosensing by bacteria: signals and membrane-based sensors. Microbiol Mol Biol Rev 63:230–262. 10066837 10.1128/mmbr.63.1.230-262.1999 PMC98963

[9] Battesti A, Majdalani N, Gottesman S 2011 The RpoS-mediated general stress response in Escherichia coli. Annu Rev Microbiol 65:189–213. doi:10.1146/annurev-micro-090110-102946. 21639793 PMC7356644

[10] Erickson DW, Schink SJ, Patsalo V, Williamson JR, Gerland U, Hwa T 2017 A global resource allocation strategy governs growth transition kinetics of Escherichia coli. Nature 551:119–123. doi:10.1038/nature24299. 29072300 PMC5901684

[11] Dai X, Zhu M, Warren M, Balakrishnan R, Patsalo V, Okano H, Williamson JR, Fredrick K, Wang YP, Hwa T 2016 Reduction of translating ribosomes enables Escherichia coli to maintain elongation rates during slow growth. Nat Microbiol 2:16231. doi:10.1038/nmicrobiol.2016.231. 27941827 PMC5346290

[12] Bremer H, Dennis PP 1996 Modulation of chemical composition and other parameters of the cell at different exponential growth rates, p 1553–1569. *In* Neidhardt FC, Curtiss R III, Ingraham JL, Lin ECC, Low KB, Magasanik B, Reznikoff WS, Riley M, Schaechter M, Umbarger HE (ed), *Escherichia coli* and *Salmonella*, 2nd ed, vol 2 American Society for Microbiology, Washington, DC.

[13] Dalbow DG, Young R 1975 Synthesis time of beta-galactosidase in Escherichia coli B/r as a function of growth rate. Biochem J 150:13–20. doi:10.1042/bj1500013. 1106403 PMC1165698

[14] Schleif R, Hess W, Finkelstein S, Ellis D 1973 Induction kinetics of the l-arabinose operon of Escherichia coli. J Bacteriol 115:9–14. 4577756 10.1128/jb.115.1.9-14.1973 PMC246203

[15] Andersson DI, Bohman K, Isaksson LA, Kurland CG 1982 Translation rates and misreading characteristics of rpsD mutants in Escherichia coli. Mol Gen Genet 187:467–472. doi:10.1007/BF00332630. 6757661

[16] Zhu M, Dai X, Wang YP 2016 Real time determination of bacterial in vivo ribosome translation elongation speed based on LacZα complementation system. Nucleic Acids Res 44:e155–e155. doi:10.1093/nar/gkw698. 27903884 PMC5175348

[17] Pedersen S 1984 Escherichia coli ribosomes translate in vivo with variable rate. EMBO J 3:2895–2898. 6396082 10.1002/j.1460-2075.1984.tb02227.x PMC557784

[18] Liang ST, Ehrenberg M, Dennis P, Bremer H 1999 Decay of rplN and lacZ mRNA in Escherichia coli. J Mol Biol 288:521–538. doi:10.1006/jmbi.1999.2710. 10329160

[19] Neidhardt FC, Magasanik B 1960 Studies on the role of ribonucleic acid in the growth of bacteria. Biochim Biophys Acta 42:99–116. doi:10.1016/0006-3002(60)90757-5. 13728193

[20] Scott M, Gunderson CW, Mateescu EM, Zhang Z, Hwa T 2010 Interdependence of cell growth and gene expression: origins and consequences. Science 330:1099–1102. doi:10.1126/science.1192588. 21097934

[21] Scott M, Klumpp S, Mateescu EM, Hwa T 2014 Emergence of robust growth laws from optimal regulation of ribosome synthesis. Mol Syst Biol 10:747. doi:10.15252/msb.20145379. 25149558 PMC4299513

[22] Maaløe O. 1979 Regulation of the protein-synthesizing machinery—ribosomes, tRNA, factors, and so on, p 487–542. *In* Goldberger RF (ed), Biological regulation and development. Plenum, New York, NY.

[23] Nath K, Koch AL 1970 Protein degradation in Escherichia coli. I. Measurement of rapidly and slowly decaying components. J Biol Chem 245:2889–2900. 4912536

[24] Klumpp S, Scott M, Pedersen S, Hwa T 2013 Molecular crowding limits translation and cell growth. Proc Natl Acad Sci U S A 110:16754–16759. doi:10.1073/pnas.1310377110. 24082144 PMC3801028

[25] Richey B, Cayley DS, Mossing MC, Kolka C, Anderson CF, Farrar TC, Record MT 1987 Variability of the intracellular ionic environment of Escherichia coli. Differences between in vitro and in vivo effects of ion concentrations on protein-DNA interactions and gene expression. J Biol Chem 262:7157–7164. 3108249

[26] Zhang G, Fedyunin I, Miekley O, Valleriani A, Moura A, Ignatova Z 2010 Global and local depletion of ternary complex limits translational elongation. Nucleic Acids Res 38:4778–4787. doi:10.1093/nar/gkq196. 20360046 PMC2919707

[27] Konopka MC, Shkel IA, Cayley S, Record MT, Weisshaar JC 2006 Crowding and confinement effects on protein diffusion in vivo. J Bacteriol 188:6115–6123. doi:10.1128/JB.01982-05. 16923878 PMC1595386

[28] Konopka MC, Sochacki KA, Bratton BP, Shkel IA, Record MT, Weisshaar JC 2009 Cytoplasmic protein mobility in osmotically stressed Escherichia coli. J Bacteriol 191:231–237. doi:10.1128/JB.00536-08. 18952804 PMC2612437

[29] Mika JT, van den Bogaart G, Veenhoff L, Krasnikov V, Poolman B 2010 Molecular sieving properties of the cytoplasm of Escherichia coli and consequences of osmotic stress. Mol Microbiol 77:200–207. doi:10.1111/j.1365-2958.2010.07201.x. 20487282

[30] Nissen P, Thirup S, Kjeldgaard M, Nyborg J 1999 The crystal structure of Cys-tRNACys-EF-Tu-GDPNP reveals general and specific features in the ternary complex and in tRNA. Structure 7:143–156. doi:10.1016/S0969-2126(99)80021-5. 10368282

[31] Yang F, Moss LG, Phillips GN Jr 1996 The molecular structure of green fluorescent protein. Nat Biotechnol 14:1246–1251. doi:10.1038/nbt1096-1246. 9631087

[32] Hink MA, Griep RA, Borst JW, van Hoek A, Eppink MH, Schots A, Visser AJ 2000 Structural dynamics of green fluorescent protein alone and fused with a single chain Fv protein. J Biol Chem 275:17556–17560. doi:10.1074/jbc.M001348200. 10748019

[33] Polikanov YS, Blaha GM, Steitz TA 2012 How hibernation factors RMF, HPF, and YfiA turn off protein synthesis. Science 336:915–918. doi:10.1126/science.1218538. 22605777 PMC3377384

[34] Milon P, Tischenko E, Tomsic J, Caserta E, Folkers G, La Teana A, Rodnina MV, Pon CL, Boelens R, Gualerzi CO 2006 The nucleotide-binding site of bacterial translation initiation factor 2 (IF2) as a metabolic sensor. Proc Natl Acad Sci U S A 103:13962–13967. doi:10.1073/pnas.0606384103. 16968770 PMC1599896

[35] Hui S, Silverman JM, Chen SS, Erickson DW, Basan M, Wang J, Hwa T, Williamson JR 2015 Quantitative proteomic analysis reveals a simple strategy of global resource allocation in bacteria. Mol Syst Biol 11:784. doi:10.15252/msb.20145697. 25678603 PMC4358657

[36] You C, Okano H, Hui S, Zhang Z, Kim M, Gunderson CW, Wang YP, Lenz P, Yan D, Hwa T 2013 Coordination of bacterial proteome with metabolism by cyclic AMP signalling. Nature 500:301–306. doi:10.1038/nature12446. 23925119 PMC4038431

[37] Li GW, Burkhardt D, Gross C, Weissman JS 2014 Quantifying absolute protein synthesis rates reveals principles underlying allocation of cellular resources. Cell 157:624–635. doi:10.1016/j.cell.2014.02.033. 24766808 PMC4006352

[38] Janssen BD, Diner EJ, Hayes CS 2012 Analysis of aminoacyl- and peptidyl-tRNAs by gel electrophoresis. Methods Mol Biol 905:291–309. doi:10.1007/978-1-61779-949-5_19. 22736012 PMC3682404

[39] Dong H, Nilsson L, Kurland CG 1996 Co-variation of tRNA abundance and codon usage in Escherichia coli at different growth rates. J Mol Biol 260:649–663. doi:10.1006/jmbi.1996.0428. 8709146

