# Supplementary material for: Slowdown of Translational Elongation in Escherichia coli under Hyperosmotic Stress
Source: mBio. 2018 Feb 13;9(1):e02375-17. doi: 10.1128/mBio.02375-17 (PMC5821080; doi:10.1128/mBio.02375-17)
Supplement: FIG S1 [file mbo001183718sf1.pdf]

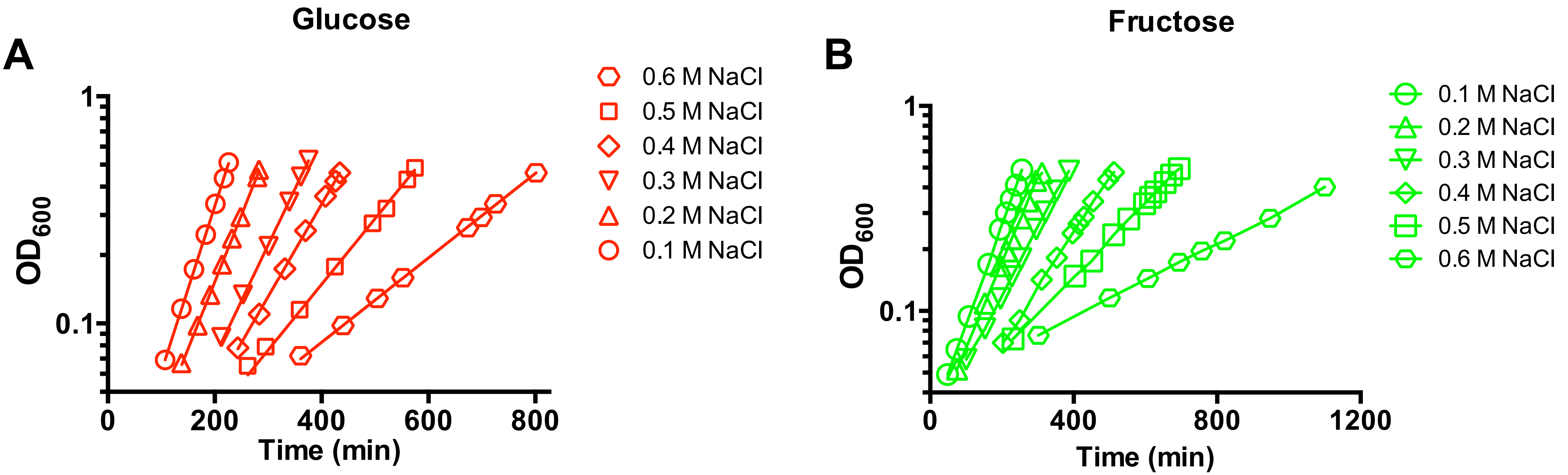
Glucose
A
Fructose
B
0.6 M NaCl
0.5 M NaCl
0.4 M NaCl
0.3 M NaCl
0.2 M NaCl
0.1 M NaCl
OD600
Time (min)
1
0.1
0
200
400
600
800
1200
