# Supplementary figures and images for: Slowdown of Translational Elongation in Escherichia coli under Hyperosmotic Stress
Source: mBio. 2018 Feb 13;9(1):e02375-17. doi: 10.1128/mBio.02375-17 (PMC5821080; doi:10.1128/mBio.02375-17)

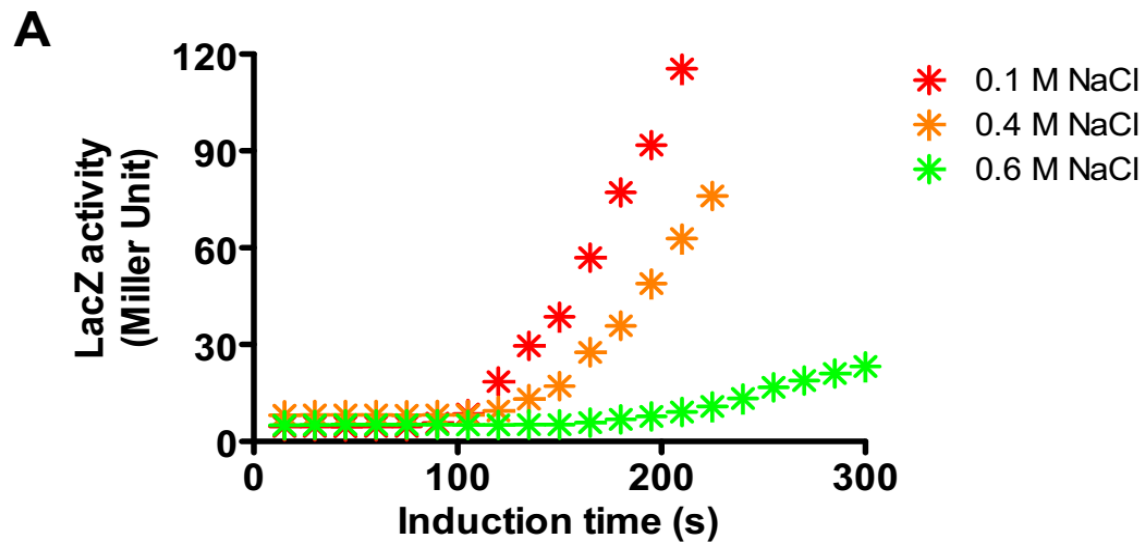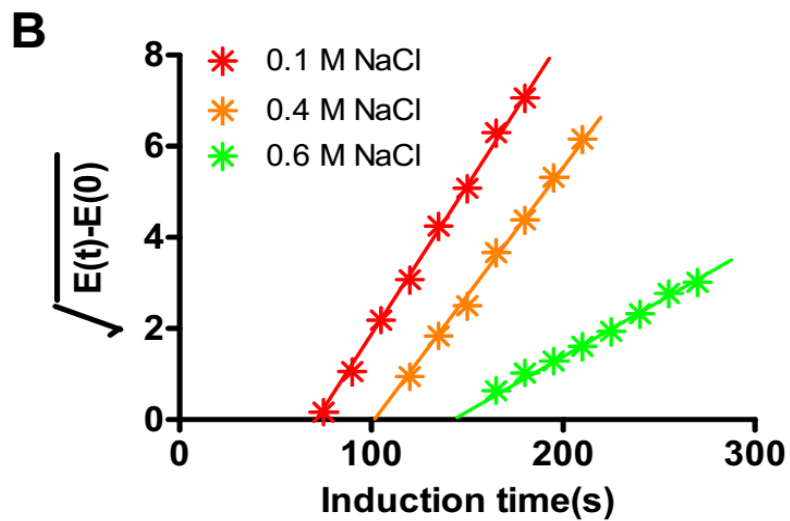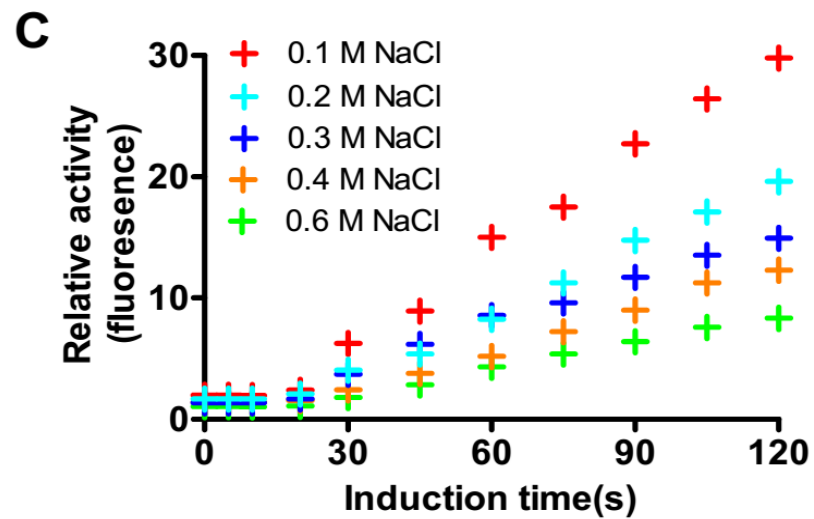

Supplement: FIG S2 [file mbo001183718sf2.pdf]

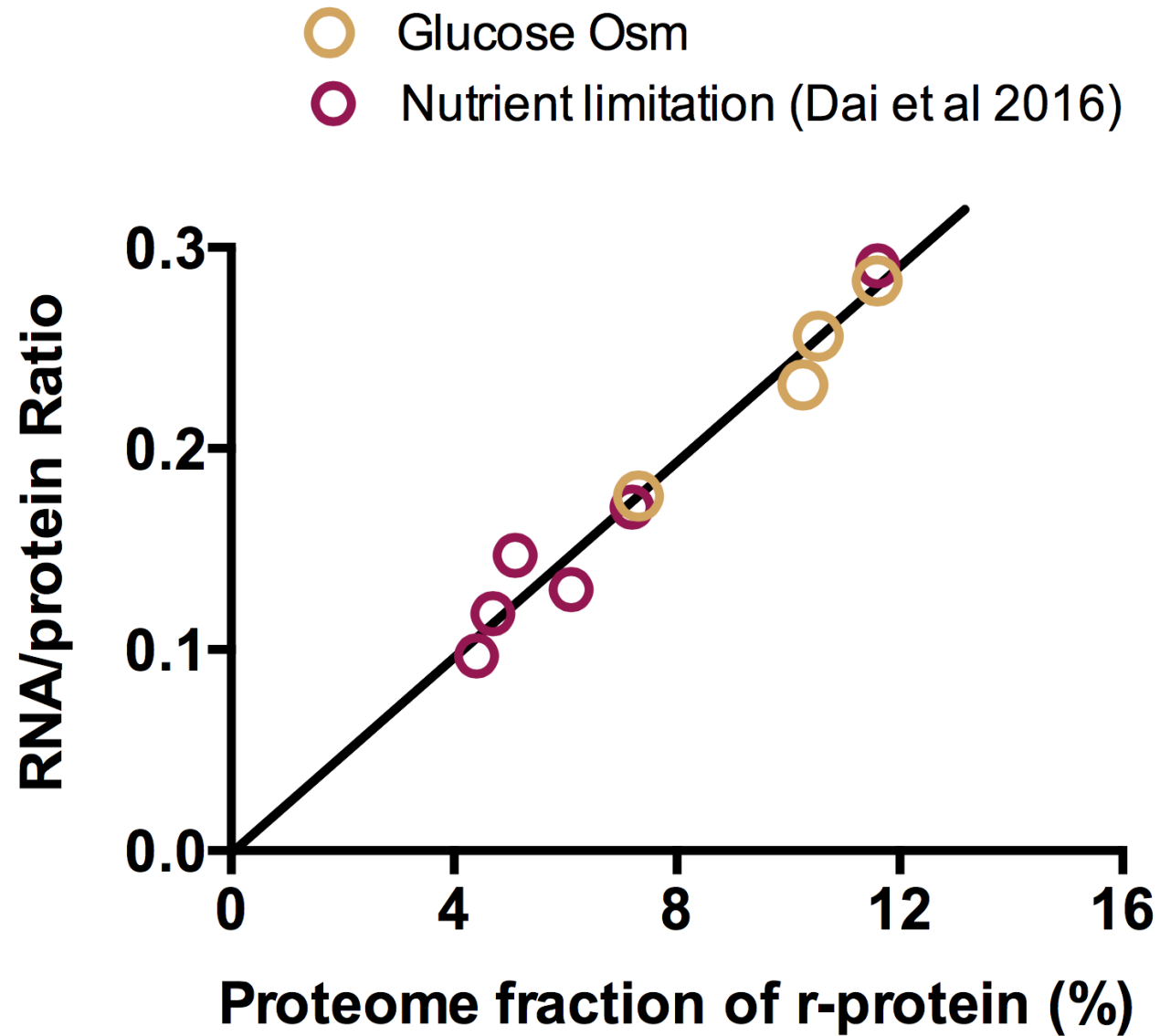

Supplement: FIG S4 [file mbo001183718sf4.pdf]

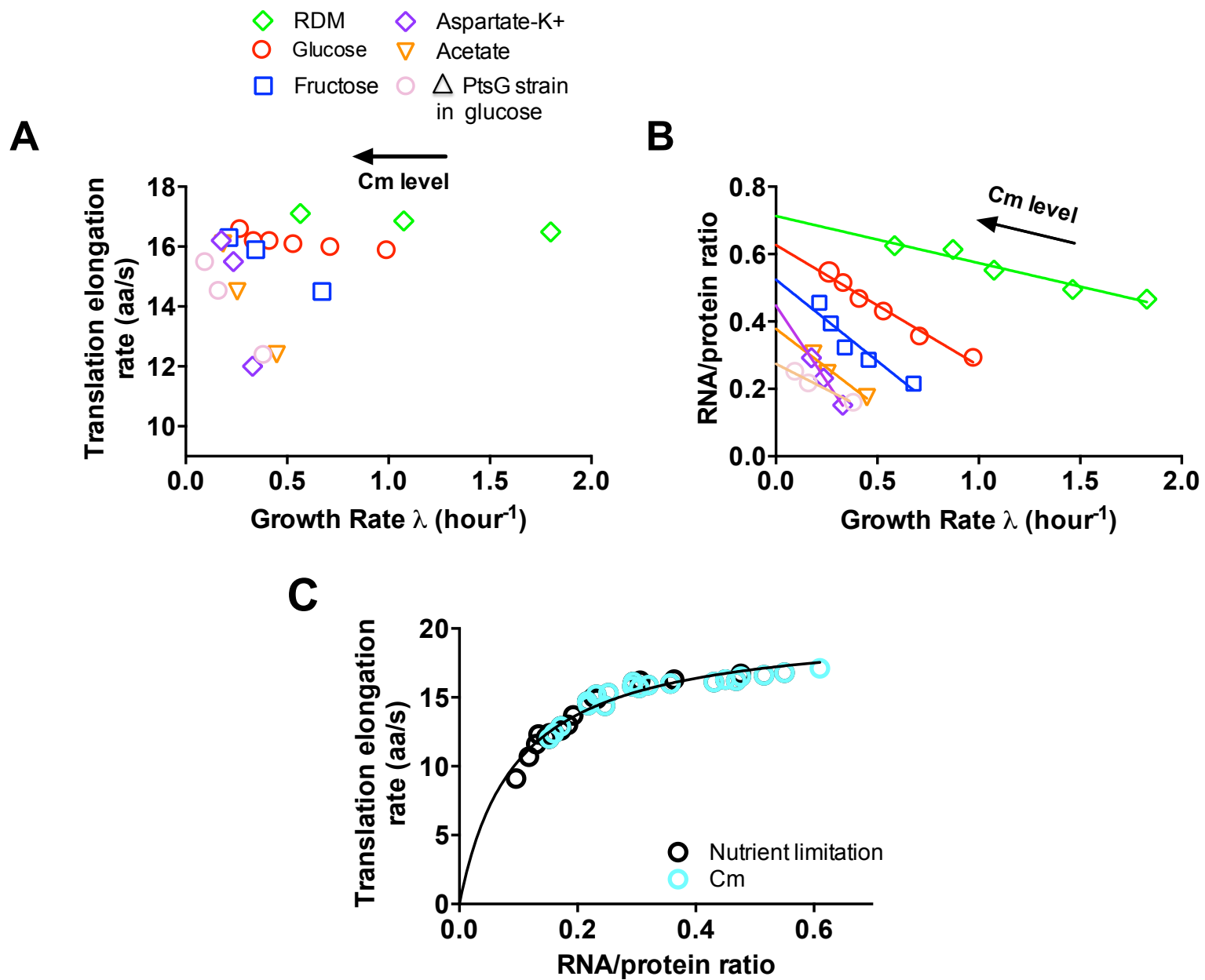

Supplement: FIG S5 [file mbo001183718sf5.pdf]

**A**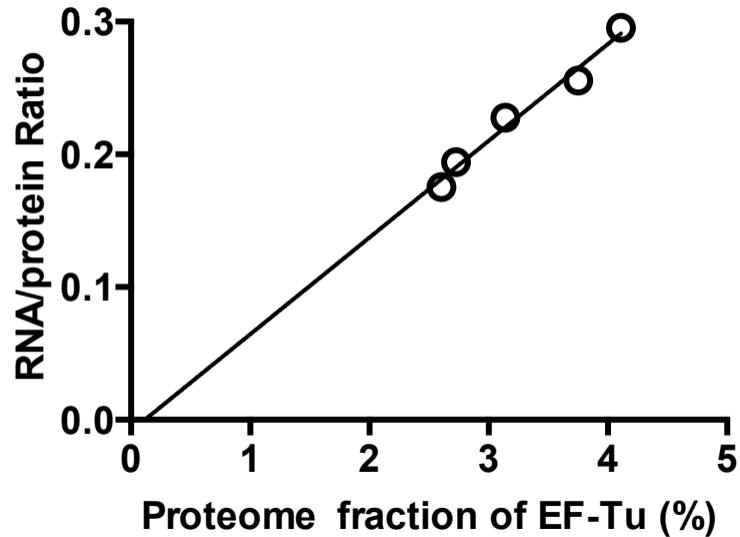**B**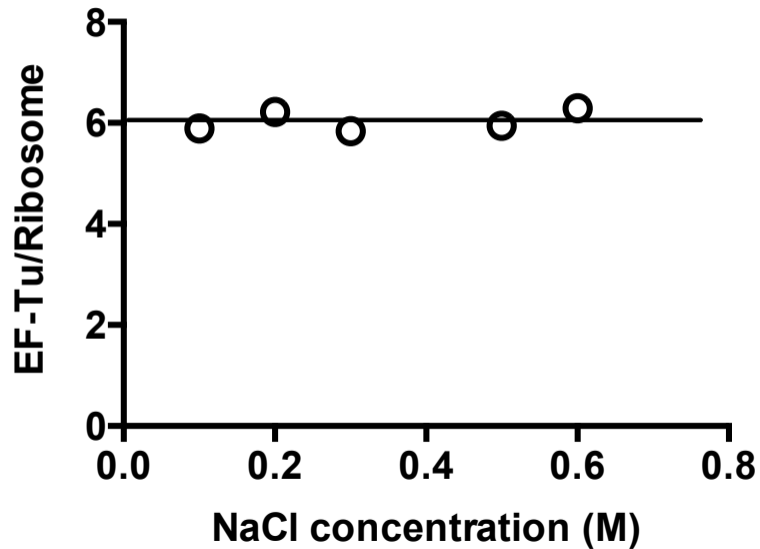

Supplement: FIG S6 [file mbo001183718sf6.pdf]

**A**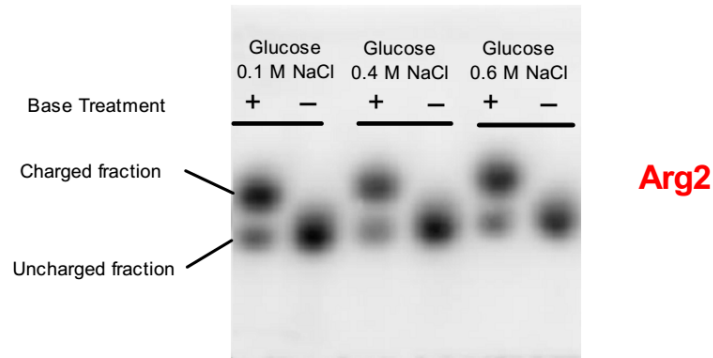**B**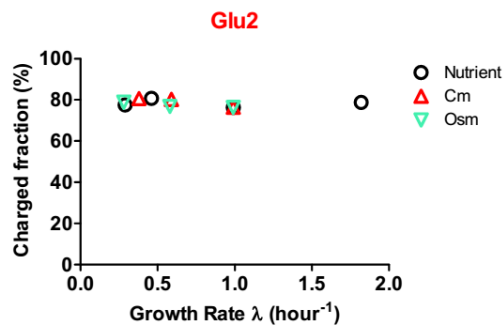**C**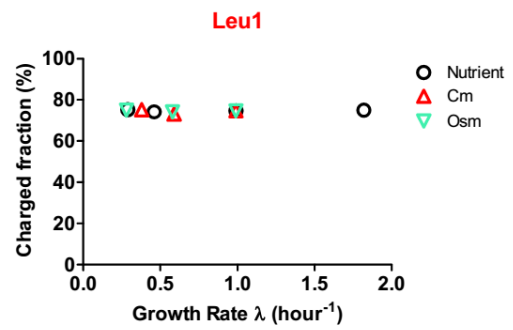**D**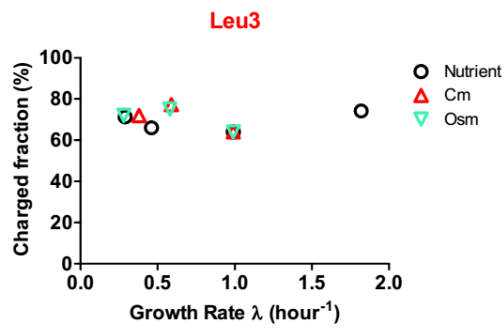**E**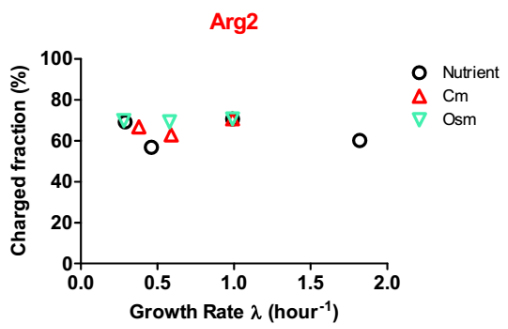**F**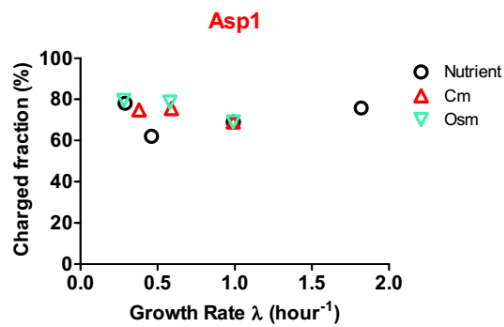**G**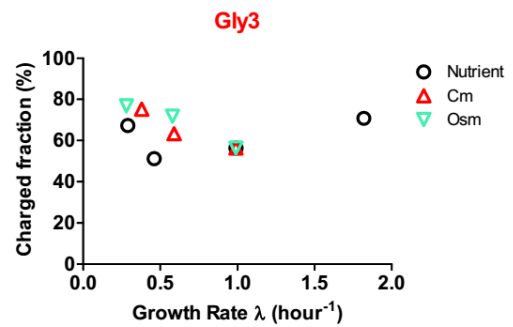

Supplement: FIG S7 [file mbo001183718sf7.pdf]

**A**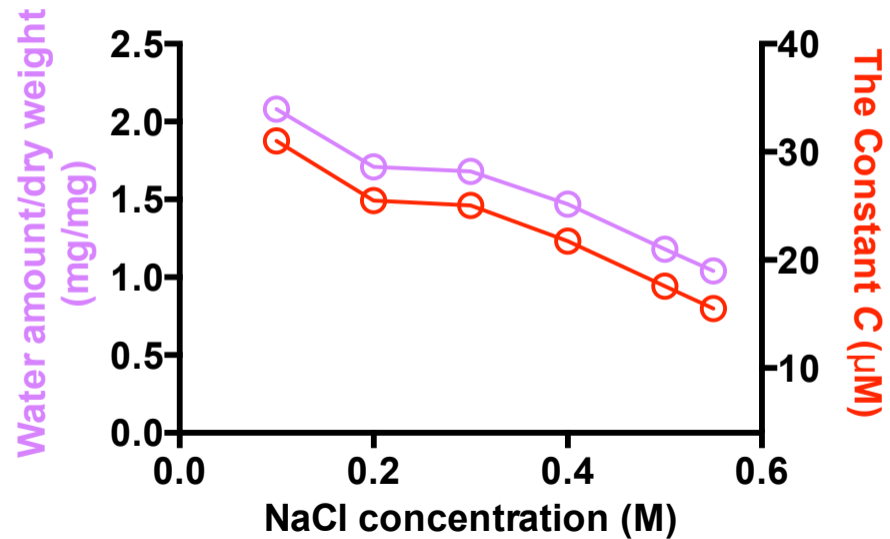**B**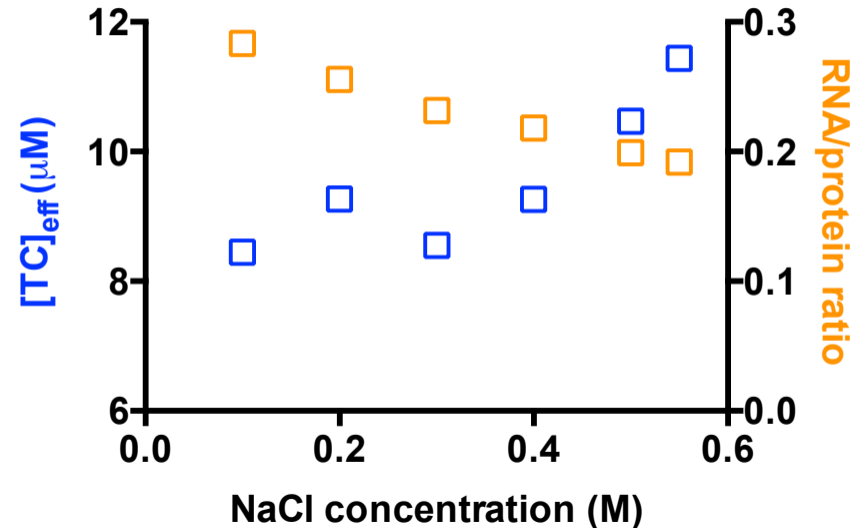

Supplement: FIG S8 [file mbo001183718sf8.pdf]

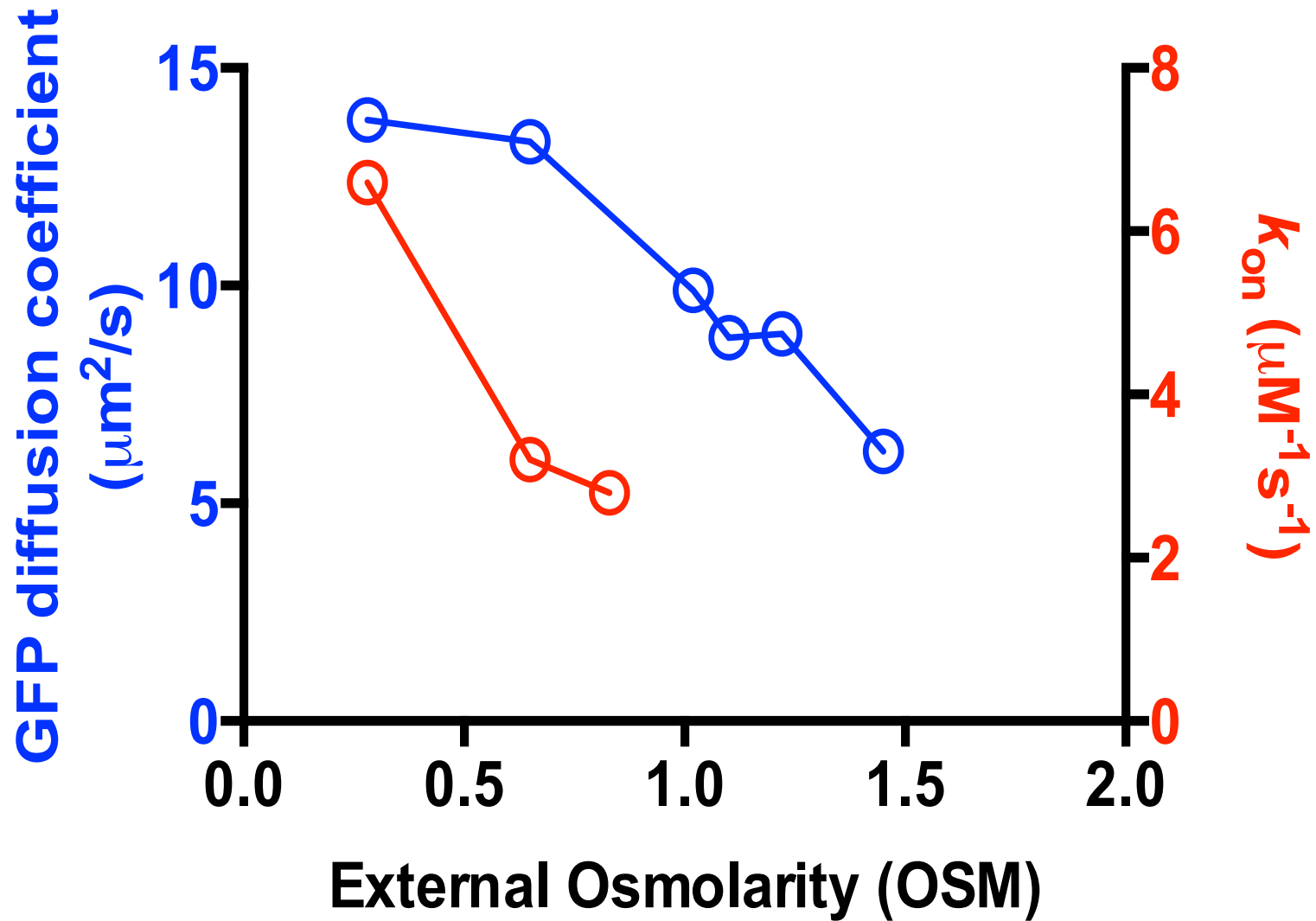

Supplement: FIG S9 [file mbo001183718sf9.pdf]
