# Supplementary material for: Slowdown of Translational Elongation in Escherichia coli under Hyperosmotic Stress
Source: mBio. 2018 Feb 13;9(1):e02375-17. doi: 10.1128/mBio.02375-17 (PMC5821080; doi:10.1128/mBio.02375-17)

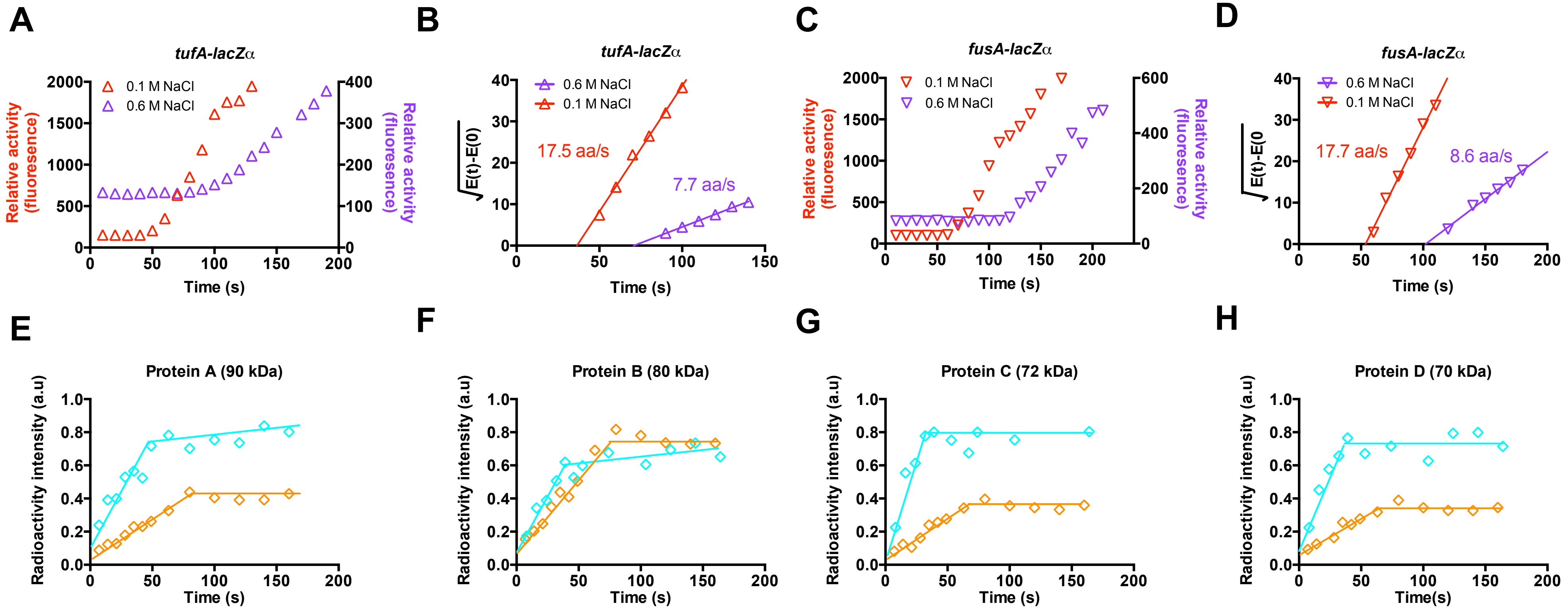

I

Conditions	GR(1/h)	Symbol	Translational elongation rate (aa/s)				
			Protein A	Protein B	Protein C	Protein D	LacZ
Glucose	0.98	◇	17.3	16.6	16.5	16.9	16.1
Glucose+0.6 M NaCl	0.26	◇	8.9	7.9	7.8	7.6	7.8

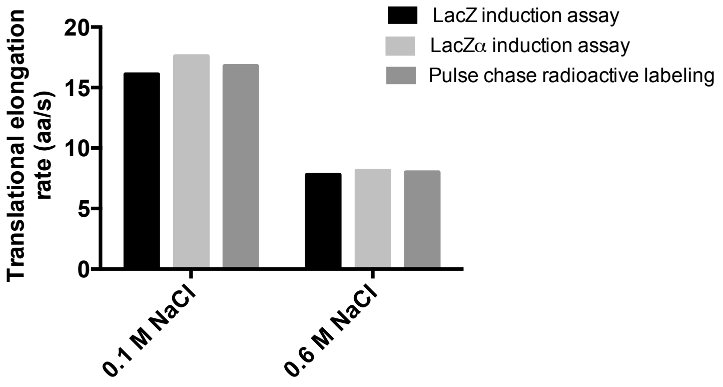

Supplement: FIG S3 [file mbo001183718sf3.pdf]
