# Supplementary material for: Slowdown of Translational Elongation in Escherichia coli under Hyperosmotic Stress
Source: mBio. 2018 Feb 13;9(1):e02375-17. doi: 10.1128/mBio.02375-17 (PMC5821080; doi:10.1128/mBio.02375-17)
Supplement: TABLE S1 [file mbo001183718st1.docx]

**Table S1A**

| **Nutrient conditions** | **NaCl (M)** | **Growth rate (1/h)** | **Translational elongation rate (aa/s)** | **Symbols** |
| --- | --- | --- | --- | --- |
| 0.2% Glucose  + 10 mM NH4Cl | 0.1 | 0.97 | 16.1 ± 0.3 |  |
|  | 0.2 | 0.82 | 14.2 ± 0.2 |  |
|  | 0.3 | 0.67 | 12.8± 0.4 |  |
|  | 0.4 | 0.56 | 11.5 ± 0.3 |  |
|  | 0.5 | 0.41 | 9.4 ± 0.1 |  |
|  | 0.55 | 0.34 | 8.5 ± 0.2 |  |
|  | 0.6 | 0.25 | 7.8 ± 0.2 |  |
|  | 0.65 | 0.12 | 6.4 ± 0.1 |  |
| 0.2% Fructose  + 10 mM NH4Cl | 0.1 | 0.67 | 14.7 ± 0.4 |  |
|  | 0.3 | 0.46 | 13.1 ± 0.2 |  |
|  | 0.4 | 0.37 | 11.6 ± 0.3 |  |
|  | 0.5 | 0.25 | 9.3 ± 0.1 |  |
|  | 0.55 | 0.17 | 8.5 ± 0.1 |  |
|  | 0.6 | 0.11 | 7.6 ± 0.3 |  |

**Table S1A. Translational elongation rate of *E. coli* under different osmolarity.** The data in glucose medium and fructose medium are shown in Figure 1B, respectively. Each value have been repeated for three times and displayed as average ± standard error.

**Table S1B**

| **Nutrient conditions** | **NaCl (M)** | **Growth rate (1/h)** | **Total RNA (µg)/OD_600_** | **Total Protein (µg)/OD_600_** | **RNA/protein** | **Symbols** |
| --- | --- | --- | --- | --- | --- | --- |
| 0.2% Glucose + 10 mM NH4Cl | 0.1 | 0.97 | 91.2 ± 1.5 | 321 ± 5.5 | 0.284 |  |
|  | 0.2 | 0.82 | 85 ± 0.8 | 328 ± 7.8 | 0.260 |  |
|  | 0.3 | 0.67 | 77 ± 0.9 | 325 ± 3.4 | 0.237 |  |
|  | 0.4 | 0.56 | 72.3 ± 2.1 | 327 ± 6.1 | 0.221 |  |
|  | 0.5 | 0.41 | 62.5 ± 0.6 | 305 ± 3.2 | 0.205 |  |
|  | 0.55 | 0.34 | 60.5 ± 0.4 | 310 ± 2.5 | 0.195 |  |
|  | 0.6 | 0.25 | 55.3 ± 1.4 | 313 ± 5.7 | 0.176 |  |
|  | 0.65 | 0.12 | 54.5 ± 0.7 | 315 ± 2.3 | 0.173 |  |
| 0.2% Fructose + 10 mM NH4Cl | 0.1 | 0.67 | 77 ± 3.5 | 349 ± 3.7 | 0.221 |  |
|  | 0.2 | 0.58 | 68.1 ± 2.3 | 330 ± 1.7 | 0.206 |  |
|  | 0.3 | 0.46 | 60.6 ± 1.1 | 353 ± 4.4 | 0.172 |  |
|  | 0.4 | 0.37 | 53.7 ± 0.6 | 321 ± 6.9 | 0.167 |  |
|  | 0.5 | 0.25 | 48 ± 0.4 | 321 ± 5.3 | 0.150 |  |
|  | 0.55 | 0.17 | 47.6 ± 1.5 | 324 ± 3.5 | 0.147 |  |
|  | 0.6 | 0.11 | 44.5 ± 0.4 | 327 ± 9.1 | 0.136 |  |

**Table S1B. RNA/protein ratio of *E. coli* under different osmolarity.** The data in glucose medium and fructose medium are shown in Figure 1C, respectively. Data of total RNA and total protein have been repeated for three times and displayed as average ± standard error.

**Table S1C**

| **Nutrient conditions** | **NaCl (M)** | **Growth rate (1/h)** | **Active ribosome fraction** | **Symbols** |
| --- | --- | --- | --- | --- |
| 0.2% Glucose + 10 mM NH4Cl | 0.1 | 0.97 | 0.89 |  |
|  | 0.2 | 0.82 | 0.94 |  |
|  | 0.3 | 0.7 | 0.93 |  |
|  | 0.4 | 0.56 | 0.93 |  |
|  | 0.5 | 0.41 | 0.89 |  |
|  | 0.55 | 0.34 | 0.87 |  |
|  | 0.6 | 0.26 | 0.76 |  |
|  | 0.65 | 0.12 | 0.46 |  |
| 0.2% Fructose + 10 mM NH4Cl | 0.1 | 0.67 | 0.88 |  |
|  | 0.3 | 0.46 | 0.87 |  |
|  | 0.4 | 0.37 | 0.8 |  |
|  | 0.5 | 0.25 | 0.74 |  |
|  | 0.55 | 0.17 | 0.59 |  |
|  | 0.6 | 0.11 | 0.45 |  |

**Table S1C. Fraction of active ribosome of *E. coli* under different osmolarity.** The data in glucose medium and fructose medium are shown in Figure 1D, respectively. The fraction of active ribosome, $f_{active}$=$N_{Rb}^{active}$*/*$N_{Rb}$= (λ**•**σ)/*k***•**(*R/P*). The data of translational elongation rate (*k*) is from Table S1A; *R/P* is from Table S1B; σ= m*_rRNA_*/(0.86**•** m*_aa_*), where m*_rRNA_* is the average molecular weight of rRNA, 1,479,384; m_aa_ is the average molecular weight of amino acid, 113.

**Table S1D**

| **Nutrient conditions** | **Cm (µM)** | **Growth rate (1/h)** | **Translational elongation rate (aa/s)** | **Symbols** |
| --- | --- | --- | --- | --- |
| 0.2% Glucose + 10 mM NH4Cl+ 0.3 M NaCl | 0 | 0.67 | 12.7 |  |
|  | 2 | 0.65 | 13.8 |  |
|  | 4 | 0.52 | 15.5 |  |
|  | 6 | 0.39 | 16.1 |  |
|  | 8 | 0.27 | 16.2 |  |
| 0.2% Glucose + 10 mM NH4Cl+ 0.4 M NaCl | 0 | 0.56 | 11.4 |  |
|  | 2 | 0.51 | 13.1 |  |
|  | 4 | 0.43 | 14.2 |  |
|  | 6 | 0.32 | 14.8 |  |
|  | 8 | 0.21 | 15.3 |  |
| 10 mM Glucose-6-phosphate + 10 mM Gluconate + 10 mM NH4Cl + 0. 4 M NaCl | 0 | 0.69 | 12.7 | * |
|  | 4 | 0.65 | 13.2 |  |
|  | 6 | 0.56 | 14.6 |  |
|  | 8 | 0.42 | 15.1 |  |

**Table S1D. Translational elongation rate of *E. coli* upon chloramphenicol inhibition at a fixed high osmolarity.** Wild type *E. coli* NCM3722 cells were grown in medium of a fixed high osmolarity supplemented with different levels of chloramphenicol. The data are shown in Figure 2A, respectively. Each value have been repeated for three times and displayed as average ± standard error.

**Table S1E**

| **Nutrient conditions** | **Cm (µM)** | **Growth rate (1/h)** | **Total RNA (µg)/OD600** | **Total Protein (µg)/OD600** | **RNA/protein** | **Symbols** |
| --- | --- | --- | --- | --- | --- | --- |
| 0.2% Glucose + 10 mM NH4Cl+ 0.3 M NaCl | 0 | 0.67 | 77 ± 0.9 | 325 ± 4.2 | 0.237 |  |
|  | 2 | 0.65 | 89.9 ± 1.5 | 312 ± 1.1 | 0.288 |  |
|  | 4 | 0.52 | 103 ± 1.6 | 305 ± 3.4 | 0.338 |  |
|  | 6 | 0.39 | 122 ± 0.4 | 298 ± 7.1 | 0.41 |  |
|  | 8 | 0.27 | 126.6 ± 1.4 | 289 ± 6.4 | 0.438 |  |
| 0.2% Glucose + 10 mM NH4Cl+ 0.4 M NaCl | 0 | 0.56 | 72.3 ± 2.1 | 327 ± 6.1 | 0.221 |  |
|  | 2 | 0.51 | 77.9 ± 1.4 | 322 ± 4.6 | 0.242 |  |
|  | 4 | 0.43 | 91.1 ± 0.8 | 309 ± 2.7 | 0.295 |  |
|  | 6 | 0.32 | 98.2 ± 1.1 | 306 ± 4.2 | 0.321 |  |
|  | 8 | 0.21 | 105.7 ± 3.5 | 285 ± 3.6 | 0.371 |  |
| 10 mM Glucose-6-phosphate + 10 mM Gluconate + 10 mM NH4Cl + 0. 4 M NaCl | 0 | 0.69 | 79.8 ± 0.4 | 331 ± 5.6 | 0.241 | * |
|  | 4 | 0.65 | 90 ± 1.9 | 336 ± 2.4 | 0.268 |  |
|  | 6 | 0.56 | 98.6 ± 2.8 | 333 ± 2.6 | 0.296 |  |
|  | 8 | 0.42 | 109.1 ± 0.6 | 320 ± 1.4 | 0.341 |  |

**Table S1E. RNA-protein ratio of *E. coli* upon chloramphenicol inhibition at a fixed high osmolarity.** Wild type *E. coli* NCM3722 cells were grown in medium of a fixed high osmolarity supplemented with different levels of chloramphenicol. The data are shown in Figure 2B, respectively. Each value have been repeated for three times and displayed as average ± standard error.
